# Effect of Ultrasonic-Assisted Enzymatic Hydrolysis on Functional Properties and Antioxidant Activity of Eri Silkworm Pupa Protein Isolate

**DOI:** 10.1155/2023/9409710

**Published:** 2023-11-29

**Authors:** Kanchana Thongrattanatrai, Rarisara Impaprasert, Worapot Suntornsuk, George Srzednicki

**Affiliations:** ^1^Department of Microbiology, King Mongkut's University of Technology Thonburi, Bangkok 10140, Thailand; ^2^Division of Food Technology, Kanchanaburi Campus, Mahidol University, Kanchanaburi 71150, Thailand; ^3^Food Science & Technology, School of Chemical Engineering, The University of New South Wales, Sydney, NSW 2052, Australia

## Abstract

*Philosamia ricini* (Eri silkworm) pupa protein isolate (EPI) was utilized to prepare pupa protein hydrolysate (EPIH) through enzymatic hydrolysis. Additionally, the isolate underwent ultrasonic treatment at 20 kHz to become ultrasound pretreated EPI (EPIU), which was then enzymatically hydrolyzed to obtain ultrasound pretreated protein hydrolysate (EPIUH). The physicochemical properties of these samples were investigated, including molecular weight, solubility, foaming and emulsion properties, water- and oil-holding capacity, antioxidant activity, and color. When compared to EPI (used as the control), EPIU exhibited a high degree of hydrolysis at 20 minutes (DH = 29.24%). At a total process time of 20 minutes, the degree of hydrolysis for EPIH, EPIU, and EPIUH was found to be 13%, 29%, and 41%, respectively. SDS-PAGE analysis indicated no difference in molecular weight between EPI and EPIU (11–75 kDa). However, the molecular weight profiles of EPIH and EPIUH were reduced (8–45 kDa), resulting in changes in protein functionalities. The high DH value contributed to the enhancement of antioxidant activity, solubility, emulsion capacity, emulsion stability, and foam capacity of the protein isolate at pH 7. Furthermore, the ultrasonic pretreatment of the protein hydrolysate increased the lightness of the protein powder by reducing the enzyme activity of the polyphenol oxidase (PPO). These results suggest that ultrasonic pretreatment of the protein hydrolysate could be applied to improve the properties of Eri silkworm pupa protein for use in the food and beverage industry, such as protein-rich beverages or salad dressings.

## 1. Introduction

Protein, commonly found in animal products, is an essential macronutrient for human consumption. Worldwide meat consumption was predicted to increase by up to 73% by 2050, compared to 2010 consumption, driven by increasing global population growth [1]. The need for novel protein sources to feed the growing world population is urgent and necessary. Insects are one of the novel protein sources with minimal environmental impact. They require less water and space and cause less pollution. Therefore, insect farming for food is an environmentally friendly alternative to traditional animal husbandry.

Recently, insects have gained more attentions in Thailand, Japan, Africa, Latin America, and other regions as an inexpensive source of good-quality proteins. They are commercially available throughout the year, both in the form of raw and processed products. Eri silkworm (*Philosamia ricini*) pupa, a by-product from the silk-reeling process, has gained increasing interest as a rich source of proteins (54.2 g/100 g of silkworm pupa dry weight) and essential amino acids (44.9 g/100 g of protein) [2]. It can be utilized for human consumption. Yang et al. [3] also reported that silkworm pupae have been used in Chinese traditional medicines since ancient times. Moreover, pharmacological studies have indicated that consumption of silkworm pupae improved human immunity, protected the human liver, and prevented breast cancer in consumers [4]. However, they are still not popular among consumers since they are not usually consumed in unprocessed forms. Recent consumer surveys in the Netherlands, Australia, and Germany suggest that introducing “invisible insects” into food products would be a good approach to enhance consumer acceptance [5]. For this reason, insect protein isolate is an alternative ingredient incorporated into foods due to its unrecognizable appearance. Additionally, protein ingredients could enhance the biological, functional, and nutritional properties of foods [6]. However, insect protein isolates have poor solubility that may significantly impact their properties when applied to food and beverage products. Therefore, several studies investigating the functional properties of insect proteins using solvents, enzymatic modification, and ultrasonic treatment have been reported [7].

Ultrasonic treatment, a green and physical processing technology, has recently been used in the food industry to enhance the functional properties of foods. Ultrasound can be classified into two categories based on the frequency range. Low-energy ultrasound (100 kHz to 1 MHz) is used for physicochemical analysis in foods [8]. High-energy ultrasound (20 to 100 kHz) is commonly applied to modify the physical and chemical properties of foods because it generates mechanical energy and high shear force through cavitation, leading to the rapid formation and collapse of gas bubbles that alter the food structure [9]. Recently, high-energy ultrasound has been used successfully used to enhance the enzymatic hydrolysis of plant proteins and improve their functional properties. Ultrasonic pretreatment increased the solubility, oil-holding capacity, and emulsifying stability of soy and jackfruit seed protein hydrolysates by unfolding their *α*-helix and *β*-turn contents [10, 11]. Furthermore, it modified the whey and *Hermetia illucens* protein structures to increase the ACE inhibitory and immunomodulatory activities of their protein hydrolysates [12, 13]. Therefore, this study is the first to report the ultrasonic-assisted enzymatic hydrolysis of the protein isolate from *Philosamia ricini* (Eri silkworm) pupae, which improved its biological and functional properties.

## 2. Materials and Methods

### 2.1. Materials

Eri silkworm pupae were obtained from Kongkiat Textile Co., Ltd. (Bangkok, Thailand) and were freeze-dried prior to grinding and defatting with hexane to produce pupa powder. Papain powder with an activity of 210 U/g was acquired from Sigma-Aldrich (USA). Other reagents, including TNBS, were of analytical grade.

### 2.2. Preparation of Eri Silkworm Pupa Protein Isolate (EPI)

Protein extraction from deoiled pupa powder was performed by adding 30 g of sample powder to 150 mL of distilled water and 0.15 g of ascorbic acid under stirring (300 rpm) at 40°C for 30 minutes [14]. The resulting extract was then centrifuged at 9,000 × *g* and 4°C for 30 minutes, and the clear supernatant was collected. Proteins in the supernatant were precipitated by adding 40 mL of cold acetone to 10 mL of the clear extract. The mixture was stirred and incubated at -20°C for 1 hour. The suspension was subsequently centrifuged at 9,000 × *g* and 4°C for 30 minutes. The resulting precipitate was collected, treated to remove residual acetone by centrifugation at 9,000 × *g* and 4°C for 30 minutes, and then freeze-dried overnight to obtain the EPI powder. The powder was evaluated for protein yield, protein molecular weights, color, functional properties, and antioxidant activity.

### 2.3. Preparation of Ultrasonic-Pretreated EPI (EPIU)

An ultrasound-treated sample was prepared by mixing 1 g of EPI powder in 20 mL of distilled water using a magnetic stirrer. The suspension was sonicated using a sonicator (VCX 130, 500, 750, Sonics & Material, Newtown, USA) at a frequency of 20 kHz for 0 minutes (control), 5 minutes, 10 minutes, 15 minutes, 20 minutes, and 25 minutes. Throughout the sonication process, the suspension was kept in an ice bath to prevent protein denaturation. Samples were collected during ultrasonic treatment to determine the degree of hydrolysis (DH) and protein molecular weights. The sample with the highest degree of hydrolysis was selected and freeze-dried to produce EPIU powder. This powder was then analyzed for color, functional properties, and antioxidant activity.

### 2.4. Preparation of Eri Silkworm Pupa Protein Hydrolysates (EPIH and EPIUH)

EPIH and EPIUH were prepared through the enzymatic hydrolysis of protein samples, EPI and EPIU, respectively, using papain. The hydrolyses were conducted by continuous shaking at 300 rpm and 40°C. The enzyme-substrate (E/S) ratio was set at 0.05 : 100 (*w*/*w*) under pH 6.5. A buffer solution was formulated by combining dibasic sodium phosphate (Na_2_HPO_4_·12H_2_O) and monobasic sodium phosphate (NaH_2_PO_4_·2H_2_O), followed by pH adjustment to achieve a value of 7.0. The protein powder was dissolved in the buffer solution, and the pH was carefully adjusted to 6.5, which corresponds to the optimal pH range for enzyme activity. Subsequently, the enzyme was introduced into the solution. All treatments were hydrolyzed for 0 minutes (control), 10 minutes, 30 minutes, 60 minutes, 90 minutes, and 120 minutes. The reactions were halted by heat treatment at 90°C for 10 minutes in a water bath. Following this, the samples were centrifuged at 9,000 × *g* at 4°C for 30 minutes. The resulting supernatants were collected to determine the degree of hydrolysis and protein molecular weights. Hydrolysates with the same degree of hydrolysis were chosen and freeze-dried to produce EPIH and EPIUH powders. These powders were then analyzed for color, functional properties, and antioxidant activity.

### 2.5. Analyses

#### 2.5.1. Degree of Hydrolysis (DH)

The degree of hydrolysis (DH) of each soluble fraction was determined using the TNBS method [15]. EPI, EPIU, EPIH, and EPIUH samples were appropriately diluted and mixed with 2 mL of 0.1 M phosphate buffer (pH 8.2) and 1 mL of 0.01% TNBS solution. The mixtures were then placed in a water bath at 50°C for 60 minutes in the dark. The reaction was terminated by adding 4 mL of 0.1 N hydrochloric acid and cooled at room temperature for 30 minutes. Absorbance was measured at 420 nm, and free amino acid content was expressed as L-leucine equivalents. The degree of hydrolysis was calculated as follows:

(1) $$\begin{array}{c} \text{DH}\left(\%\right)=\left(\frac{L_{t}–L_{0}}{L_{\max}–L_{0}}\right)\times 100, \end{array}$$

where *L*_0_ represents the free amino acid content of the protein sample before hydrolysis (mg/g protein), *L*_*t*_ represents the free amino acid content of the protein sample after hydrolysis (mg/g protein), and *L*_max_ represents the total free amino acids in the protein sample after acid hydrolysis with 6 M HCl at 100°C for 24 hours.

#### 2.5.2. Protein Molecular Weight

The molecular weights of EPI, EPIU, EPIH, and EPIUH were determined using SDS-PAGE following the modified procedure of Yi et al. [16]. The samples with an appropriate concentration were electrophoresed at a constant 80 V in a stacking gel containing 5% (*w*/*v*) acrylamide and a separating gel containing 12% (*w*/*v*) acrylamide for 90 minutes. Electrophoresis was considered complete when the bromophenol blue marker had migrated to a distance of 1 to 1.5 cm from the bottom of the gel. The gel was then stained with Coomassie Blue R-250 for 30 minutes with continuous shaking. Finally, the gel was destained until a clear background was achieved. The molecular weight of the protein was estimated using a 3.5 to 245 kDa molecular weight protein standard maker (TriColor Broad Protein Ladder, biotechrabbit, Germany).

#### 2.5.3. Color

The color of the EPI, EPIU, EPIH, and EPIUH samples was determined using the Color Quest XE colorimeter (Hunter Lab, USA). Sample colors were measured and expressed in terms of CIE, *L*^∗^ (lightness), *a*^∗^ (redness), and *b*^∗^ (yellowness), with the instrument standardized using standard color tiles, namely, white (*X*: 82.18, *Y*: 87.18, and *Z*: 94.03) and green (*X*: 18.69, *Y*: 24.69, and *Z*: 21.05), under the D65/10° illuminant mode and SPIN LAV observer. Subsequently, the sample color was measured by placing it into a quartz cell (transmission cell), filling at least 3/4 of the cell volume, and then measuring the color value in the CIE Lab^∗^ system through three repeated measurements.

#### 2.5.4. Antioxidant Activity

The antioxidant activity of EPI, EPIU, EPIH, and EPIUH samples was determined based on their scavenging ability against DPPH (2,2-diphenyl-1- picrylhydrazyl) free radicals, expressed as IC_50_ concentration, following the method of Murakami et al. [17] with a slight modification. The reaction mixture consisted of 100 *μ*L of the sample solution at various concentrations (62.5 to 1,000 *μ*g/mL) and 100 *μ*L of 0.1 mM DPPH [18] dissolved in 95% ethanol. All samples were incubated for 30 minutes in the dark and then measured at a wavelength of 517 nm using a spectrophotometer. The DPPH scavenging activity was calculated using the following formula:

(2) $$\begin{array}{c} \text{Scavenging activity}\left(\%\right)=\left(\frac{1-A_{\text{sample}}}{A_{\text{control}}}\right)\times 100, \end{array}$$

where *A*_sample_ is the absorbance of the sample and *A*_control_ is the absorbance of the control. The concentration that provided a 50% inhibition value (IC_50_) was determined by plotting the sample concentrations against scavenging activity on a graph.

#### 2.5.5. Functional Properties of Protein

*(1) Solubility*. The solubility of EPI, EPIU, EPIH, and EPIUH samples was determined following the methodology outlined by Gresiana et al. [19]. For each pH, the sample was suspended in distilled water until a final concentration of 4 mg/mL was achieved. The pH of the solution was adjusted from pH 2 to 9 using 0.1 N NaOH or 0.1 M HCl, while the suspension was stirred (200 rpm) at ambient temperature for 2 hours. The precipitate was separated by centrifugation at 9,000 × *g* for 15 minutes. The protein content of the supernatant was then determined using Lowry's method [20]. Complete solubility (100%) was assumed when no residue was observed after centrifugation. All experiments were performed in triplicates.

*(2) Water-Holding Capacity*. Water-holding capacity (WHC) of EPI, EPIU, EPIH, and EPIUH samples was determined following the modified method of Diniz and Martin [21]. A sample of 0.5 g was dispersed in 20 mL of distilled water, and the pH was adjusted to 7.0. The suspension was stirred using a shaker at 540 rpm for 30 minutes. Subsequently, the suspension was centrifuged at 8,000 × *g* for 15 minutes. The weight of the resulting precipitate was measured, and the weight difference was calculated. The results were expressed as grams of absorbed water per gram of the sample (g_water_/g_sample_).

*(3) Oil-Holding Capacity*. Oil-holding capacity (OHC) of the protein samples was determined following the method of Haque and Mozaffar [22]. Ten milliliters of vegetable oil was added to 0.5 g of the sample and mixed for 30 seconds using a vortex mixer. The dispersion was subsequently centrifuged at 8,000 × *g* for 15 minutes. The weight of the resulting precipitate was measured, and the weight difference was calculated. The results were presented as grams of absorbed oil per gram of the sample (g_oil_/g_sample_).

*(4) Emulsifying Properties*. Emulsifying properties of EPI, EPIU, EPIH, and EPIUH samples were assessed using the method described by Wu et al. [23]. The sample was dispersed in distilled water (1% *w*/*v*, with pH adjusted to 7.0). Subsequently, 15 mL of the dispersion was homogenized with 15 mL of vegetable oil at a speed of 18,000 rpm for 1 minute. After allowing it to stand for 10 minutes, the volumes of the individual layers were recorded. Emulsion stability (ES) was evaluated by heating the emulsion at 80°C for 30 minutes, followed by measuring the volumes of the individual layers after standing for 10 minutes. Emulsion activity (EA) and stability (ES) were calculated using the following formulas:

(3) $$\begin{array}{c} \text{Emulsion activity}\left(\%\right)=\left(\frac{V_{e}}{V}\right)\times 100,\\ \text{Emulsion stability}\left(\%\right)=\left(\frac{V_{30}}{V_{e}}\right)\times 100, \end{array}$$

where *V* is the total volume of tube contents (mL), *V*_*e*_ is the volume of the emulsified layer (mL), and *V*_30_ is the volume of the emulsified layer after heating (mL).

*(5) Foaming Properties*. Foam capacity (FC) and foam stability (FS) of EPI, EPIU, EPIH, and EPIUH samples were determined using the method outlined by Guo et al. [24]. A protein solution (1% *w*/*v*, with pH adjusted to 7.0) of twenty milliliters was homogenized using a high-shear homogenizer mixer at a speed of 14,000 rpm for 2 minutes. The total volume was measured at both time 0 and 30 minutes after homogenization. The foaming capacity and foam stability were calculated as follows:

(4) $$\begin{array}{c} \text{Foaming capacity}\left(\%\right)=\left[\frac{V_{0}–V}{V}\right]\times 100,\\ \text{Foam stability}\left(\%\right)=\left(\frac{V_{30}}{V_{0}}\right)\times 100, \end{array}$$

where *V* is the volume before whipping (mL), *V*_0_ is the volume after whipping (mL), and *V*_30_ is the volume after standing (mL).

### 2.6. Statistical Analysis

All data are presented as means ± standard deviation. Statistical analysis was conducted using STATISTICA v.10.0 for one-way analysis of variance (ANOVA), and the differences in means between the samples were determined using the Duncan test. *p* values below 0.05 were considered statistically significant.

## 3. Results and Discussion

### 3.1. Protein Extraction

Proteins in the deoiled pupa powder were extracted using water and precipitated with acetone, yielding 58.8% with a protein purity of 90.3%. Similarly, Gresiana et al. [19] also employed acetone to extract proteins from crickets (*Gryllus mitratus*) with a yield of 65% and a protein purity of 99%.

### 3.2. Degree of Hydrolysis

**Figure 1:**
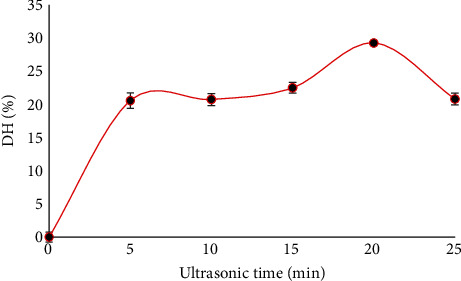
Effect of ultrasonic time on degree of hydrolysis of protein isolate.

The degree of hydrolysis (DH) value of EPI was determined using the TNBS method, which quantifies free amino groups. The effects of ultrasonic time on the DH of EPI are depicted in Figure 1. The DH value initially increased with an increasing ultrasonic time up to 20 minutes (approximately 29%), but it decreased with further increase in ultrasonic pretreatment time. This reduction could be attributed to protein reassembly, which decreased the exposure of free amino acids. This result aligns with findings from the study by Wu et al. [12]. The ultrasonic pretreatment time of 20 minutes was selected for enzymatic hydrolysis as it provided the highest DH value.

**Figure 2:**
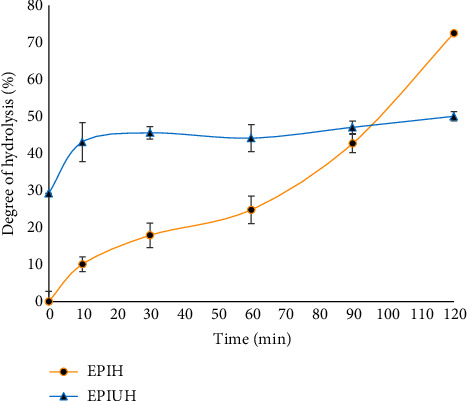
The degree hydrolysis of protein hydrolysate and ultrasound-pretreated protein hydrolysate collected at different times.

Figure 2 illustrates the DH value of EPIUH compared with EPIH at the same hydrolysis time. EPIUH achieved the highest DH after 30 minutes of hydrolysis at 50.04%, whereas EPIH reached a similar DH of 50.04% after 95 minutes of hydrolysis. Notably, EPIUH consistently exhibited higher DH values than EPIH up to 95 minutes. This suggests that the ultrasonic pretreatment was effective in promoting the enzymatic hydrolysis of EPI. This phenomenon can be attributed to the cavitation effect of ultrasound, which disrupts the protein's structure and exposes enzyme-binding sites. Jin et al. [25] reported reduced thermodynamic parameters (*E*_*a*_, Δ*H*, and Δ*S*) for gluten meal hydrolysis due to ultrasonic pretreatment. Similar results were observed in whey protein hydrolysis by Wu et al. [12], where the reduced energy required to initiate a chemical reaction facilitated better interaction with papain. The outcomes of this study demonstrate that ultrasonic pretreatment enhances the efficiency of Eri silkworm hydrolysis and reduces the required protein hydrolysis time.

### 3.3. Molecular Weight

**Figure 3:**
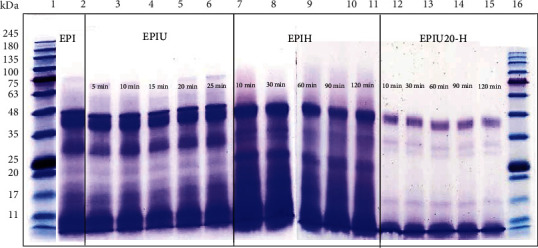
The SDS-PAGE profile of proteins: molecular weight standard (lanes 1 and 18), EPI (lane 2), EPIU (lanes 3 to 7), EPIH (lanes 8 to 12), and EPIU20-H (lanes 13 to 17).

Figure 3 depicts the molecular weights of EPI, EPIU, EPIH, and EPIUH. The molecular weight of EPIU is indicated in lanes 3 to 7. The protein bands of EPIU exhibited no discernible difference from those of EPI. Gülseren et al. [26] reported that the molecular weight of milk protein, as determined by SDS-PAGE, showed no significant difference between untreated and ultrasound-pretreated (12.5 W at 50% amplitude for 2 minutes) samples. Similar outcomes were also observed by O'Sullivan et al. [27] and Zisu et al. [28], who found no discrepancy in the molecular weights of proteins such as bovine gelatin, fish gelatin, egg white protein, soy protein isolate, pea protein isolate, rice protein isolate, and whey protein concentrate between the control and ultrasound-pretreated samples. Conversely, Jambrak et al. [29] noted that ultrasonic pretreatment (20 kHz for 15 minutes) could lead to a reduction in the molecular weight of whey protein isolate.

The prominent protein bands around 46 and 20 kDa vanished after 10 to 120 minutes of hydrolysis for EPI and EPIU, respectively (lanes 8 to 17, Figure 3). This effect might be attributed to the unfolding of the protein structure due to ultrasonic pretreatment, which subsequently enhances enzymatic hydrolysis. Consequently, EPI, EPIU (at 20 minutes of ultrasound treatment), EPIH (at 95 minutes of enzymatic hydrolysis), and EPIUH (at 20 minutes of ultrasound treatment and 30 minutes of enzymatic hydrolysis) were selected for further tests to assess protein functional properties and antioxidant activity.

### 3.4. Color

Color is an important property of food products that directly influences consumer acceptance or rejection.  Table 1 shows the colors of EPI, EPIU, EPIH, and EPIUH expressed as lightness (*L*^∗^), redness (*a*^∗^), and yellowness (*b*^∗^) values. Results show that all samples had a light brown color. Color formation was most likely due to enzymatic browning reactions, as polyphenol oxidase (PPO) in the protein isolates and hydrolysates oxidized the o-diphenols to o-quinones, leading to polymerization, which produces the brown pigment (melanin). Effects of different treatments are illustrated in Table 1. *L*^∗^ increased in the following order: EPI < EPIU < EPIH < EPIUH, whereas the reverse occurred with the *a*^∗^ value, which might be due to the reduction of PPO activities by ultrasonic treatment and enzymatic hydrolysis. Cavitation in ultrasonic treatment has been reported to accelerate the chemical breakdown of susceptible particles such as enzymes in the samples [30]. They also found a significant increase in *L*^∗^ values and a decrease in the *a*^∗^ value of the apple juice treated with ultrasounds.

### 3.5. Antioxidant Activity

Antioxidants are substances that prevent or slow down damage induced by free radicals in our bodies. Antioxidant activity values determined by DPPH are shown in Table 1. The IC_50_ value of all treated samples (EPIU, EPIH, and EPIUH) was lower than that of EPI, which is similar to the findings by Mintah et al. [13]. The high antioxidant activity of EPIUH could be due to the effects of sonication aiding the unfolding of both isolate and hydrolysate samples, exposing sulfhydryl and hydrophobic groups of the samples to free radicals, leading to enhanced antioxidant activity. Additionally, enzymatic hydrolysis enhanced by cavitation in ultrasonic treatment resulted in the presence of lower molecular weight proteins with hydrophobic and sulfhydryl groups of amino acids, improving antioxidant activity. Wang et al. [11] found that ultrasonic pretreatment of *β*-conglycinin (7S) and glycinin (11S) from soy protein hydrolysates increased free sulfhydryl content. Guerra-Almonacid et al. [31] reported that antioxidant activity of *Erythrina edulis* increased after ultrasonic pretreatment and hydrolysis. However, all samples had significantly higher antioxidant activity values than Trolox (9.28 *μ*g/mL). Thus, the treatment of EPI by ultrasound and/or enzymatic hydrolysis increased antioxidant activity, which may be useful for various food products. From the results, ultrasonic pretreatment can promote enzymatic hydrolysis to degrade protein molecules into small protein molecules with the capacity to donate electrons and react with free radicals to convert them into more stable products [32].

From the experimental results, it was observed that the molecular weight of the protein after sonication and hydrolysis was 8-45 kDa. It can be noticed that samples with a high percentage of hydrolysis exhibit a higher ability to resist free radicals compared to samples with higher molecular weights and longer peptide chains (EPIUH vs. EPI and EPIU). This is because peptides with lower molecular weights expose more side chain residues, facilitating reactions between peptides and free radicals, thus enhancing DPPH radical scavenging activity [33]. In all cases, hydrolyzed proteins with a molecular weight below 3 kDa demonstrated an increased antioxidant potential. Peptides with notable antioxidant activity typically consist of shorter chains containing 4–6 residues. As a result, the secondary structure is likely a minor contributing factor to the antioxidant properties of peptides due to their relatively low molecular weight [34]. This aligns with the research conducted by Razali et al. [35], who found that the lower molecular weight of cobia skin gelatin hydrolysate (3-10 kDa) is believed to possess stronger DPPH radical scavenging activity.

### 3.6. Functional Properties

#### 3.6.1. Solubility

**Figure 4:**
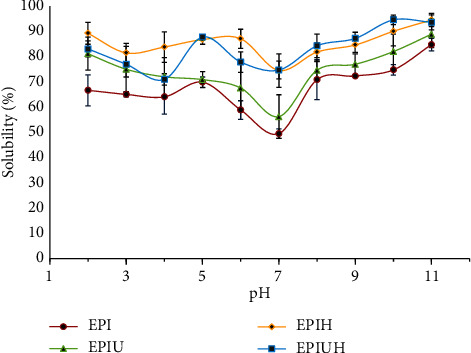
Solubility of untreated and treated protein isolate at pH 2-11.

Results of EPI, EPIU, EPIH, and EPIUH solubility are presented as a function of pH ranging from 2.0 to 11.0 in Figure 4. In this study, the solubility of the samples pretreated by ultrasound and hydrolysis was higher than that of the control (*p* < 0.05). Solubility increased in the order of EPI < EPIU < EPIH and EPIUH at pHs 7.0-11.0. This enhancement might be attributed to the high-intensity ultrasound, which induced structural and conformational changes in the protein, thus exposing hydrophilic amino acids to water. Enzymatic hydrolysis of the protein could result in even higher solubility than ultrasonic pretreatment alone. Increased solubility could also stem from the reduction in the secondary structure of proteins or the unfolding of protein molecules, leading to the release of smaller peptides. The subsequent rise in ionization of amino and carboxyl groups can promote interactions with water molecules [15]. Consistent with the study by Mintah et al. [13], protein derived from the black soldier fly (*Hermetia illucens*) exhibited elevated solubility when subjected to ultrasonic treatment prior to enzymatic digestion. The highest solubility was observed in proteins pretreated with ultrasonic waves, followed by enzymatically digested proteins. Comparatively lower solubility was noted in proteins treated solely with ultrasonic waves or those that underwent no pretreatment process. Jambrak et al. [29] also reported increased protein solubility of soy protein concentrates after ultrasonic treatment with 20 kHz compared to untreated samples. Yanjun et al. [36] observed a significant rise in the solubility of milk protein concentrates from 35.78% to 88.30% following 20 kHz ultrasonic pretreatment for 5 minutes. Thus, the solubility of EPI could be enhanced through ultrasonic pretreatment combined with enzymatic hydrolysis.

#### 3.6.2. Water-Holding Capacity

The water-holding capacity of foods is described as the ability of the food structure to retain water within the protein network, which significantly influences texture properties such as mouthfeel, juiciness, and tenderness of food products [37]. Several factors contribute to water-holding ability, including amino acid profiles, charges, conformation, pH, temperature, ionic strength, and hydrophobicity of the protein [38]. The water-holding capacity of all hydrolysates (EPIH and EPIUH) was significantly lower (*p* < 0.05) than that of the unhydrolyzed samples (EPI and EPIU) (Table 2). This reduction might be attributed to enzymatic hydrolysis, which can lead to the disruption of the protein structure and consequently reduce water-holding capacity. Meinlschmidt et al. [39] reported that the water-binding capacity of soy protein decreased from an initial value of 2.6 mL/g to 0.2 mL/g after hydrolysis with Alcalase. According to dos Santos et al. [40], a high solubility can lead to a decrease in the water-holding capacity of Bluewing Searobin (*Prionotus punctatus*) protein hydrolysate. Resendiz-Vazquez et al. [10] found that ultrasonic-assisted enzymolysis of jackfruit seed protein significantly decreased its water-holding capacity. However, the water-holding capacity of EPIU was higher than that of EPI. This increase in water-holding capacity may be attributed to the release of a large number of available polar groups during ultrasonic treatment, which could more easily interact with water. Li et al. [41] investigated the effects of ultrasonic pretreatment on the water-holding capacity of chicken meat batters and found that the samples treated with ultrasound exhibited significantly higher water-holding capacity than the control. Similarly, Zhang et al. [42] reported that ultrasound-treated gluten protein displayed higher water-holding capacity compared to untreated samples.

#### 3.6.3. Oil-Holding Capacity

Oil-holding capacity refers to the amount of oil retained within a protein structure, playing a vital role in enhancing the mouthfeel and flavor retention of food products. Ultrasonic treatment of EPI (EPIU sample) resulted in a 22% increase in oil-holding capacity compared to EPI (Table 2). Cavitation during ultrasonic treatment exposes nonpolar side chains and hydrophobic groups that can bind to hydrocarbon moieties of oil, leading to an increase in oil-holding capacity. Yu et al. [43] reported that ultrasonic treatment (20 kHz for 18 minutes) improved the oil-holding capacity of the *Mytilus edulis* protein isolate. Similar results were reported by Zhang et al. [42], showing that the oil-holding capacity of wheat gluten protein increased from 0.58 g/g (untreated) to 1.59 g/g (28/40/50 kHz for 10 minutes). In contrast, the oil-holding capacity of all hydrolysates (EPIH and EPIUH) was significantly lower than that of the unhydrolyzed samples (EPI and EPIU) (*p* < 0.05). The relatively low molecular weights of EPIH and EPIUH may contribute to these diminished values, resulting in a larger specific surface area. As a consequence, this leads to the generation of an increased number of lipophilic sites, thereby exposing nonpolar groups or hydrophobic surfaces to the solvent. Noman et al. [44] described that hydrolysis of Chinese sturgeon protein with papain decreased its oil-holding capacity because high solubility indicates the presence of smaller protein molecules, leading to a decrease in oil absorption.

#### 3.6.4. Emulsifying Properties

The emulsifying properties of a protein are related to its amphipathic nature, solubility, diffusion rate, and other characteristics. The results of emulsion capacity and emulsion stability tests for EPI, EPIU, EPIH, and EPIUH are shown in Table 2. The emulsion capacity increased in the order of EPI < EPIH and EPIUH < EPIU. Ultrasonic treatment could lead to a higher emulsion capacity compared to samples from enzymatic hydrolysis (EPIH and EPIUH). This result indicates that ultrasonic pretreatment improved molecular flexibility and surface hydrophobicity by unfolding the protein structure. Previous reports also found that ultrasonic treatment improved emulsion capacity and emulsion stability of bovine gelatin, fish gelatin, and egg white protein [27], sunflower protein isolate [45], and *Mytilus edulis* protein isolate [43]. The lower values of emulsion capacity for EPIH and EPIUH may be related to the small peptides produced by enzymatic hydrolysis. A reduction in the molecular weight of proteins enhances the diffusion rate due to their inability to undergo rearrangement and form an elastic protein film at the interface between oil and water. Another study also showed that the emulsifying properties of hydrolysates were closely related to the degree of hydrolysis, where a high degree of hydrolysis decreased emulsifying properties [46].

Regarding emulsion stability, EPI had significantly lower emulsion stability (69%) than the other samples, which might be due to its larger molecular size. The decrease in molecular size of ultrasound-treated or hydrolyzed samples resulted in increased emulsion stability [47]. O'Sullivan et al. [27] also reported that large droplet sizes exhibited gravitational separation with a cream layer that was responsible for the instability of the emulsion. Moreover, the emulsion stability of EPIU was not significantly different from that of EPIH and EPIUH (*p* ≥ 0.05). The results from the present study suggest that EPIU, EPIH, and EPIUH may be applied for colloidal food formulations to improve their emulsion stability.

#### 3.6.5. Foaming Properties

Foam capacity and foam stability of EPI, EPIU, EPIH, and EPIUH at pH 7.0 are shown in Table 2. In this study, foam capacity increased in the following order: EPI < EPIU < EPIH and EPIUH. Thus, the cavitation of ultrasonic treatment might unfold protein molecules, causing them to migrate rapidly, open up, and reposit at the air-water interface to decrease tension at the interface. Jambrak et al. [48] also reported that ultrasonic treatment of whey protein suspensions with 20 kHz improved foam capacity. Furthermore, enzymatic hydrolysis of the protein can provide a higher foam capacity. This might be due to the reduction of molecular weight leading to increased molecular flexibility and greater exposure of hydrophobic groups, resulting in an increased diffusion rate at the air-water interface. Mintah et al. [13] found that ultrasonically treated *Hermetia illucens* larva protein hydrolysate showed excellent foam capacity. The decrease in molecular weight of proteins contributes to increased solubility and plays a significant role in their foaming behavior for several reasons. Firstly, proteins with reduced molecular weight tend to have a higher surface area-to-volume ratio, which promotes better dispersion and solubility in aqueous solutions. This enhanced solubility allows proteins to interact more effectively with water molecules and form stable colloidal solutions. Additionally, proteins with lower molecular weight exhibit improved surface activity, making them more capable of reducing surface tension at interfaces. This surface activity is crucial for the formation and stabilization of foams. The reduced molecular weight facilitates the adsorption of proteins at the air-water interface, forming a stable protein film that can trap air bubbles and create foam structures. Furthermore, proteins with lower molecular weight are often more flexible and dynamic, enabling them to undergo conformational changes more readily. This flexibility allows them to unfold and reorient at interfaces, leading to increased interactions with air or oil molecules and consequently better foam stability. Overall, the decreased molecular weight of proteins enhances their solubility and contributes to their foaming behavior by promoting improved dispersion, surface activity, and conformational adaptability at interfaces.

Regarding foam stability, the EPIU was more stable than EPI, EPIH, and EPIUH. For foam stability, some larger protein components are required; however, only a few large peptides were found in EPIH and EPIUH, as shown by the DH value and SDS-PAGE, which led to weaker foam stability. According to Hall et al. [49], the size of hydrolyzed proteins mainly contributed to rapid foam formation. Nevertheless, the hydrolyzed proteins may not be good at forming the protein-protein interactions that provide a high stability of foam. As mentioned earlier, enzymatic hydrolysis of the samples improved the foaming capacity because of increased molecular flexibility of the proteins leading to a greater exposure of hydrophobic groups. However, low molecular weight peptides are not able to form a cohesive film at the interface and cause foam instability.

## 4. Conclusions

In this study, various treatments of protein isolate from Eri silkworm (*Philosamia ricini*) pupae demonstrated the enhancement of functional properties and antioxidant activity, which were caused by the unfolding of the protein structure and reduction in protein molecular size through ultrasonic treatment and enzymatic hydrolysis. The improved solubility of the protein isolate promoted the foaming capacity and emulsion capacity of EPIU, EPIH, and EPIUH. Furthermore, treatment of EPI decreased *L*^∗^ by modifying the PPO activity, which may enhance consumer acceptance. The highest antioxidant activity was found in the EPIUH sample. Therefore, it may be suitable for the fortification of functional or therapeutic foods. However, ultrasonic treatment and enzymatic hydrolysis decreased water- and oil-holding capacity, which may not be suitable for improving food texture in bakery products and ground meal formulations. Moreover, additional studies should be conducted regarding the toxicity of the isolates and their effectiveness as a food ingredient in clinical trials. The data obtained in this study may assist in the selection of treatments to modify the properties of protein isolates for various food applications.

## Figures and Tables

**Table 1:** Antioxidant activity and color value of untreated and treated protein isolate.

Treatment	IC_50_ (*μ*g/mL)	*L* ^∗^ value	*a* ^∗^ value	*b* ^∗^ value
Trolox	9.28 ± 0.26d	—	—	—
EPI	157.32 ± 0.44a	76.88 ± 0.24d	4.39 ± 0.05a	18.92 ± 0.14b
EPIU20	91.06 ± 0.75b	82.45 ± 0.30c	2.91 ± 0.04b	16.52 ± 0.04c
EPIH95	91.52 ± 0.32b	84.60 ± 0.28b	2.76 ± 0.07c	19.33 ± 0.31a
EPIU20-H30	84.09 ± 0.45c	85.18 ± 0.13a	2.64 ± 0.02d	18.72 ± 0.06b

Values in the same column having the same letter for each parameter are not significantly different at a confidence level of 95%. EPI: control/protein isolate; EPIU20: 20-minute ultrasound-treated protein isolate; EPIH95: acid-digested protein isolate for 95 minutes; EPIU20-H30: the protein isolate was treated by ultrasound for 20 minutes and acid-digested for 30 minutes.

**Table 2:** Functional properties of untreated and treated protein isolate.

Treatment	Water-holding capacity (g_water_/g_sample_)	Oil-holding capacity (g_oil_/g_sample_)	Emulsion capacity (%)	Emulsion stability (%)	Foam capacity (%)	Foam stability (%)
EPI^∗^	0.33 ± 0.07a	4.25 ± 0.08b	31.82 ± 6.43c	69.44 ± 4.81b	95.00 ± 0.00c	92.37 ± 0.11b
EPIU20^∗∗^	0.53 ± 0.15a	5.19 ± 0.53a	63.64 ± 0.00a	81.90 ± 6.60a	105.37 ± 5.56b	96.60 ± 1.61a
EPIH95^∗∗∗^	0.00 ± 0.25b	2.95 ± 0.32c	53.33 ± 1.05b	84.80 ± 1.88a	141.48 ± 2.57a	77.15 ± 2.09d
EPIU20-H30^∗∗∗∗^	0.00 ± 0.14b	2.59 ± 0.07c	54.55 ± 0.00b	83.33 ± 0.00a	143.98 ± 6.05a	80.26 ± 1.45c

Values in the same column having the same letter for each parameter are not significantly different at a confidence level of 95%. ^∗^EPI: control/protein isolate; ^∗∗^EPIU20: 20-minute ultrasound protein isolate; ^∗∗∗^EPIH95: acid-digested protein isolate for 95 minutes; ^∗∗∗∗^EPIU20-H30: the protein isolate was treated by ultrasound for 20 minutes and acid-digested for 30 minutes.

## Data Availability

The research data used to support the findings of this study are available from the corresponding author upon request.
